# Curves with prescribed rational points

**DOI:** 10.1007/s40993-026-00783-6

**Published:** 2026-09-18

**Authors:** Katerina Santicola

**Affiliations:** Department of Mathematics, King’s College London, Strand, London, WC2R 2LS UK

**Keywords:** Diophantine equations, Rational points, Primary: 11D41, 11G30, Secondary: 11D61, 11G05

## Abstract

Given a smooth curve $C/{\mathbb {Q}}$ with genus ≥2, we know by Faltings’ Theorem that $C({\mathbb {Q}})$ is finite. Here we ask the reverse question: given a finite set of rational points $S\subseteq {\mathbb {P}}^n({\mathbb {Q}})$, does there exist a smooth projective curve $C/{\mathbb {Q}}$ contained in ${\mathbb {P}}^n$ such that $C({\mathbb {Q}})=S$? We answer this question in the affirmative by providing an effective algorithm for constructing such a curve.

## Introduction

The genus *g* of a smooth projective curve $C/{\mathbb {Q}}$ determines the possibilities for its set of rational points. If g=0, then $C({\mathbb {Q}})=\emptyset$ or $C({\mathbb {Q}})$ is infinite. If g=1, then $C({\mathbb {Q}})=\emptyset$, or by the Mordell–Weil theorem, the set $C({\mathbb {Q}})$ can be given the structure of a finitely generated abelian group. Mazur’s torsion theorem [9] restricts the possible torsion subgroups of elliptic curves. As a consequence, for example, there are no genus 1 curves $C/{\mathbb {Q}}$ with exactly 11 rational points. For g≥2, Faltings’ theorem [3] asserts that $C({\mathbb {Q}})$ is finite. The Uniformity conjecture of Caporaso et al. [2] predicts that $\# C({\mathbb {Q}})$ is uniformly bounded as *C* ranges through curves $C/{\mathbb {Q}}$ of fixed genus g≥2.

It is therefore natural to ask about the possible restrictions on $\# C({\mathbb {Q}})$ as *C* ranges through all curves $C/{\mathbb {Q}}$ of all possible genera g≥2. Poonen [10] has shown that every value of $\#C({\mathbb {Q}})$ is possible (in fact, that every value of #C(K) is possible for any global field *K*). In this paper, we show that there are no restrictions on the set $C({\mathbb {Q}})$ itself, beyond finiteness.

### Theorem 1

Let n≥2. Given any finite set of rational points $S\subset {\mathbb {P}}^n({\mathbb {Q}})$, there exists a smooth projective curve $C/{\mathbb {Q}}$ contained in ${\mathbb {P}}^n$, of genus g≥2, such that $C({\mathbb {Q}})=S$.

Our proof gives an effective algorithm for computing the curve *C* from the given set of rational points *S*.

### Remark 2

If in the statement of Theorem 1 we had not asked that *C* be a projective curve, then one could simply use Bertini’s theorem [7, Corollary 6.7.(2)] to find a smooth curve *C* of large genus passing through all points of *S*. Then, by cutting away a hyperplane containing the finitely many points outside *S*, produce a quasiprojective curve whose rational points are exactly *S*.

## Outline of proof

In Sect. 3 we will introduce Theorem 5, which constructs curves with prescribed rational points in ${\mathbb {A}}^2$. Then we will prove Theorem 1 from Theorem 5 by taking complete intersections of the curves constructed in Theorem 5, generalising the construction to ${\mathbb {P}}^n$.

Sections 4 and 5 are dedicated to the proof of Theorem 5. The proof of Theorem 5 builds on earlier constructions due to Gajović [4] and the current author [11]. Let $\mathcal {P}_R$ denote the perfect powers of the ring *R*:

$$\begin{aligned} \mathcal {P}_R:=\{ a^m: a\in R,m\ge 2\}. \end{aligned}$$

They show that for a finite set of perfect powers $S\subset \mathcal {P}_R$, for $R={\mathbb {Z}}$ and $R={\mathbb {Q}}$ respectively, there is a polynomial $f_S(X)\in {\mathbb {Z}}[X]$ such that $f_S(R)\cap \mathcal {P}_R = S$. We will show how the construction of the polynomial $f_S$ in those papers can be adapted to give a construction of a suitable ‘superelliptic’ curve *C* in our context.

By a *superelliptic curve* *C* [5, Definition 1] we mean a smooth projective curve in ${\mathbb {P}}^2$ associated to the affine model given by

$$\begin{aligned} C:\; Y^m=f(X), \end{aligned}$$

where *f* is separable of degree d≥3 and m≥2 is an integer. When m=d, the genus *g* of the curve is g=(d-1)(d-2)/2; thus if d≥4, then g≥2 and so $C({\mathbb {Q}})$ is finite by Faltings’ theorem [3].

Section 4 is dedicated to constructing a polynomial $f_{S,\mathcal {B}}(X)$ and degree *d*, corresponding to the given set of points *S* and some finite set $\mathcal {B}\subset {\mathbb {C}}$, such that the superelliptic curve $C:\; Y^d=f_{S,\mathcal {B}}(X)$ satisfies $S\subseteq C({\mathbb {Q}})$. In Sect. 5 we prove Theorem 5, and most importantly Lemma 15, where we prove that $C({\mathbb {Q}})\subseteq S$. A key step in the proof of Lemma 15 is relating the rational points of *C* to the rational points of an associated elliptic curve.

## Proof of Theorem 1 from Theorem 5

We begin by addressing the case |S|=1 separately. Our general argument will assume |S|>1.

### Lemma 3

Let n≥2. Given any rational point $P\in {\mathbb {P}}^n({\mathbb {Q}})$, there exists a smooth projective curve $C/{\mathbb {Q}}$ contained in ${\mathbb {P}}^n$, of genus g≥2, such that $C({\mathbb {Q}})=\{P\}$.

### Proof of Lemma 3

Consider the hyperelliptic curve over ${\mathbb {Q}}$ with affine model

$$\begin{aligned} C:Y^2=X^5-2. \end{aligned}$$

We verify in Magma [1] that the rational points on the Jacobian, $J({\mathbb {Q}})$, form a group of rank 0 with trivial torsion subgroup. Since $C({\mathbb {Q}})$ injects into $J({\mathbb {Q}})$, we conclude that *C* has no rational points other than the point at infinity. *C* can be embedded in ${\mathbb {P}}^n$ for any n≥2, and a suitable rational change of coordinates will send the point *P* to the point at infinity on *C*. □

We now introduce the definition of an *acceptable* set of points.

### Definition 4

Let $S=\{P_1,\dots ,P_r\}\subset {\mathbb {A}}^n({\mathbb {Q}})$ be a finite set of distinct rational points in affine space. We say that *S* is *acceptable* if the following conditions are satisfied:

- for all *i*, *j*, we have $x_i(P_j)\ne 0$,
- for all *i*, *j*, *u*, *v* with i≠u or j≠v, we have $x_i(P_j)\ne x_u(P_{v})$,
- for all *i*, *j*, we have $x_i(P_j)\in {\mathbb {Z}}$,

where the $x_i$ are the usual coordinate functions.

For any acceptable set of points $S\subset {\mathbb {A}}^2({\mathbb {Q}})$, we construct in Theorem 5 a suitable superelliptic curve *C* such that $C({\mathbb {Q}})=S$.

### Theorem 5

Given an acceptable set $S\subset {\mathbb {A}}^2({\mathbb {Q}})$ with |S|>1, and a finite subset $\mathcal {B} \subset {\mathbb {C}}$, there exists a separable polynomial $f_{S,\mathcal {B}}(X)\in {\mathbb {Q}}[X]$ of some degree d≥4, not vanishing at any point in $\mathcal {B}$, such that $C({\mathbb {Q}})=S$, where *C* is the superelliptic curve associated to the affine model

$$\begin{aligned} C \; : \; Y^d=f_{S,\mathcal {B}}(X). \end{aligned}$$

We now show how Theorem 1 follows from Theorem 5. We begin with Lemma 6 which shows that an arbitrary set of points can be transformed to fit the requirements of Theorem 5.

### Lemma 6

Let $S=\{P_1,\dots ,P_r\}\subset {\mathbb {A}}^n({\mathbb {Q}})$ be a finite set of distinct non-zero points. Then there exists a change of coordinate matrix $A\in \operatorname {GL}_n({\mathbb {Q}})$ such that $\{AP_1,\dots ,AP_r\}\subset {\mathbb {A}}^n({\mathbb {Q}})$ is acceptable.

### Proof

We write $M_{n \times n}$ for the affine space of dimension $n^2$ considered as the space of n×n matrices. Consider the following subvarieties of $M_{n \times n}$:

- $$U=\{ A \in M_{n \times n} : \det (A) =0\}$$,
- $$V_{i,j}=\{ A \in M_{n \times n} : x_i(AP_j)=0\}$$ with $$1 \le i \le n$$ and $$1 \le j \le r$$,
- $W_{i,j,u,v}=\{ A \in M_{n \times n} : x_i(A P_j) = x_u(A P_{v})\}$ with $1 \le i, u \le n$ and $1 \le j , v \le r$ and either i≠u or j≠v.

It is easy to see that these are all proper subsets of $M_{n \times n}$ which are closed in the Zariski topology. As the rational points on $M_{n \times n}$ are Zariski dense, it follows that there is some $A \in M_{n \times n}({\mathbb {Q}})$ that does not lie on any of the *U*, $V_{i,j}$, $W_{i,j,u,v}$. We can scale *A*, if necessary, to ensure integrality. □

Combining these Lemmas, we are able to deduce Theorem 1 from Theorem 5.

### Proof of Theorem 1

Let *S* be a finite set of rational points in ${\mathbb {P}}^n({\mathbb {Q}})$. If |S|=1, apply Lemma 3. Otherwise, let |S|=r>1. By finiteness of this set, we can always find a hyperplane on which none of the points lie. After a suitable change of coordinates, all the points are non-zero and lie in an affine chart. By Lemma 6 we may assume that the set $S=\{P_1,\ldots ,P_r\}\subset {\mathbb {A}}^n({\mathbb {Q}})$ is acceptable.

For $$2 \le j \le n$$, let

$$\begin{aligned} S_j:=\{(x_1(P_1),x_j(P_1)) \, , \, (x_1(P_2),x_j(P_2)) \, , \, \ldots \, , \, (x_1(P_r),x_j(P_r))\} \; \subset \; {\mathbb {A}}^2({\mathbb {Q}}). \end{aligned}$$

Since *S* is acceptable, the $S_j$ are also acceptable sets for all $2\le j \le n$, with $|S_j|=|S|=r>1$. We construct sequences of finite subsets of ${\mathbb {C}}$:

$$\begin{aligned} \mathscr {B}_2 \subset \mathscr {B}_3 \subset \cdots \subset \mathscr {B}_n \end{aligned}$$

and a sequence of separable polynomials $f_2,f_3,\ldots ,f_n \in {\mathbb {Q}}[X]$ as follows. We start by letting $\mathscr {B}_2=\emptyset$. We let $f_2=f_{S_2,\mathscr {B}_2}$ constructed as in Theorem 5. For the inductive step of the construction, let $\mathscr {B}_j$ be the union of $\mathscr {B}_{j-1}$ and the set of complex roots of $f_{j-1}$, and we let $f_j=f_{S_j,\mathscr {B}_j}$. Let $C \subset {\mathbb {P}}^n$ be the projective closure of the affine variety

$$\begin{aligned} V\left( X_2^d - f_2(X) \, , \, X_3^{d} - f_3(X) \, , \, \cdots \, , \, X_n^{d}-f_n(X) \, \right) \; \subset {\mathbb {A}}^n. \end{aligned}$$

It follows from Theorem 5 that $C({\mathbb {Q}})=\{P_1,P_2,\dots ,P_r\}$. Smoothness of *C* follows from applying the Jacobian criterion. In the affine patch, we use that $f_2,\ldots ,f_n$ are separable and have pairwise distinct complex roots, and on the hyperplane at infinity, we use that the $f_j$ are of degree *d*. □

## Constructing $f_{S,\mathcal {B}}$

From now on we assume that |S|=r>1 and that $S=\{(a_1,b_1),\dots ,(a_r,b_r)\}\subset {\mathbb {A}}^2({\mathbb {Q}})$ is acceptable. Set

$$\begin{aligned} d=18r+3, \end{aligned}$$

and let

$$\begin{aligned} m:=\prod _{j<k}^r (a_j-a_k). \end{aligned}$$

Let

$$\begin{aligned} S'=\{(a_i,b_i^d)\mid (a_i,b_i)\in S\}, \end{aligned}$$

and let $$L(X)\in {\mathbb {Q}}[X]$$ be the Lagrange interpolation polynomial whose graph interpolates the points in $S^{\prime }$,

$$\begin{aligned} L(X):=\sum _{j=1}^r b_j^d\prod _{\begin{array}{c} 1\le k \le r\\ k\ne j \end{array}}\frac{X-a_k}{a_j-a_k}. \end{aligned}$$

### Lemma 7

Let $S=\{(a_1,b_1),\dots ,(a_r,b_r)\}\subset {\mathbb {A}}^2({\mathbb {Q}})$ be an acceptable set of points of size |S|=r. Let *n* be any integer such that n≥r. Then there exists an irreducible polynomial

$$\begin{aligned} h(X)=\sum _{i=0}^n \alpha _i X^i \in {\mathbb {Q}}[X] \end{aligned}$$

satisfying the following conditions:

(i) $$h(a_i)=b_i^{d}$$ for all $$(a_i,b_i)\in S$$,
(ii) *h*(*X*) has degree *n*,
(iii) $$m \cdot h(X) \in {\mathbb {Z}}[X]$$.

### Proof of Lemma 7

Let

$$\begin{aligned} b(X)=\prod _{\begin{array}{c} i=1 \end{array}}^r(X-a_i)\in {\mathbb {Z}}[X]. \end{aligned}$$

Since *S* is acceptable, $b_j\ne 0$ for all *j*, so *L*(*X*) and *b*(*X*) are coprime. Choose $c(X)\in {\mathbb {Z}}[X]$ to be monic and coprime to *L*(*X*) of degree n-r, and let

$$\begin{aligned} R(X,Y)=L(X)+b(X)c(X)Y \in {\mathbb {Q}}(Y)[X]. \end{aligned}$$

Since *R*(*X*, *Y*) is degree 1 in *Y*, any proper factorisation in ${\mathbb {Q}}(Y)[X]$ would require one of the factors to be degree 0 in *Y*. Thus *R*(*X*, *Y*) is irreducible in ${\mathbb {Q}}(Y)[X]$ since *L*(*X*) is coprime to *b*(*X*)*c*(*X*). By Hilbert’s irreducibility theorem [12, Proposition 3.3.5], there is a thin set $A\subset {\mathbb {P}}^1({\mathbb {Q}})$ such that if t∉A, then *R*(*X*, *t*) is irreducible in ${\mathbb {Q}}[X]$. The integers ${\mathbb {Z}}\subset {\mathbb {A}}^1({\mathbb {Q}})$ are not thin [12, Proposition 3.4.3], so it follows that we may choose a non-zero integer y∉A. Choose any such *y* and let h(X)=R(X,y). Then *h*(*X*) will satisfy the conditions above. □

We continue with our construction of $f_{S,\mathcal {B}}$. Apply Lemma  7 with n=6r+3 to produce *h*(*X*) of degree *n*. Let *N* be the radical of *m*,

$$\begin{aligned} N:=\prod _{v_p(m)>0}p. \end{aligned}$$

With $S=\{(a_1,b_1),\dots , (a_r,b_r)\}\subset {\mathbb {A}}^2({\mathbb {Q}})$ as before, let

$$\begin{aligned} g(X,t):=tN^6\prod _{i=1}^r(X-a_i)^6+1 \in {\mathbb {Q}}[X,t]. \end{aligned}$$

### Proposition 8

Let *g*(*X*, *t*), *h*(*X*) be as above. Given a finite set of values B⊂C, the zeroes of $g(X, t_0)$ and $(h(X)-1)g(X,t_0)+1$ lie outside of $\mathcal {B}$ for all but finitely many values of $t_0\in {\mathbb {C}}$.

### Proof

Every $\alpha \in \mathcal {B}$ is a root of $g(X,t_0)$ for at most one value $t_0\in {\mathbb {C}}$, since *g*(*X*, *t*) is linear in *t*. The same reasoning applies to the polynomial (h(X)-1)g(X,t)+1. □

### Lemma 9

Let *g*(*X*, *t*) be as above. For all but finitely many primes *p*,

$$\begin{aligned} (h(X)-1)g(X,p)+1\in {\mathbb {Q}}[X] \end{aligned}$$

is separable.

### Proof of Lemma 9

First, because *h*(*X*) is irreducible, its discriminant $\Delta _h$ is non-zero, and $v_p(\Delta _h)=0$ for all but finitely many primes *p*. For these primes *p* such that $v_p(\Delta _h)=0$, *h*(*X*) is separable modulo *p*. Now rewrite the polynomial (h(X)-1)g(X,t)+1 as

1 $$\begin{aligned} (h(X)-1) g(X,t)+1 = h(X) + t H(X) = \sum _{i=0}^{6r+n} c_i X^i, \end{aligned}$$

where *H*(*X*) is some polynomial in ${\mathbb {Q}}[X]$. For all but finitely many values of *p*, we can specialise *g*(*X*, *t*) at t=p so that the valuations of the coefficients are

$$\begin{aligned} v_p(c_i)={\left\{ \begin{array}{ll} 0 &  0 \le i \le n,\\ 1 &  n+1 \le i \le 6r+n. \end{array}\right. } \end{aligned}$$

Thus the Newton polygon of the polynomial (1) consists of two segments of lengths *n* and 6*r* and slopes 0 and 1/6*r* respectively. By the theory of Newton polygons [6, Proposition 7.2.3], the polynomial (1) with t=p will factor as the product of two irreducible polynomials $F_1(X),F_2(X)$ in ${\mathbb {Z}}_{p}[X]$:

$$\begin{aligned} (h(X)-1)g(X,p)+1 \; =\; F_1(X)F_2(X) \end{aligned}$$

corresponding to the two line segments of the Newton polygon. Here $F_1(X)$ has degree *n* and its roots have valuation 0, and $F_2(X)$ has degree 6*r* and its roots have valuation 1/6*r*. In particular, $F_1$, $F_2$ are coprime. Moreover, $F_1 \equiv h(X) \mod p$. However *h*(*X*) is separable modulo *p*, and thus $F_1$ is separable in ${\mathbb {Z}}_p[X]$, for all but finitely many *p*. The second factor $F_2$ is irreducible because it is pure of slope 1/6*r*. This means their product $F_1(X)F_2(X)$ is separable for all but finitely many primes *p*. □

With all of this in mind, we may choose a prime ℓ that satisfies a list of conditions.

### Proposition 10

Given a finite set $\mathcal {B}\subset {\mathbb {C}}$, we can choose a prime ℓ such that

(i) the zeroes of g(X,ℓ) and (h(X)-1)g(X,ℓ)+1 lie outside of $\mathcal {B}$
(ii) (h(X)-1)g(X,ℓ)+1 is separable,
(iii) (ℓ,N)=1,
(iv) $$\ell \equiv 5\mod 12$$.

### Proof of Proposition 10

This is always possible by Proposition 8, Lemma 9 and Dirichlet’s theorem on arithmetic progressions. □

## Proof of Theorem 5

Let ℓ be a prime chosen as in Proposition 10. Let

$$\begin{aligned} g(X):=g(X,\ell )=\ell N^6 \prod _{i=1}^r(X-a_i)^6+1. \end{aligned}$$

Let

$$\begin{aligned} f_{S,\mathcal {B}}(X):=g(X)((h(X)-1)g(X)+1), \end{aligned}$$

and let *C* be the projective curve associated to the affine equation

$$\begin{aligned} C: Y^d=f_{S,\mathcal {B}}(X). \end{aligned}$$

We note that for all $1\le i \le r$,

2 $$\begin{aligned} f_{S,\mathcal {B}}(a_i)=h(a_i)=b_i^d \end{aligned}$$

since $g(a_i)=1$, so $S \subseteq C({\mathbb {Q}})$ as required. Moreover,

3 $$\begin{aligned} \deg f_{S,\mathcal {B}}(X)=2\deg g(X)+\deg h(X)=18r+3=d. \end{aligned}$$

### Lemma 11

The polynomial $f_{S,\mathcal {B}}(X)$ is separable.

### Proof of Lemma 11

Note that *g*(*X*) and (h(X)-1)g(X)+1 are coprime in ${\mathbb {Q}}[X]$, so it is enough to show that each is separable. We have shown the latter is separable in Lemma 9, and *g*(*X*) is irreducible by examining the Newton polygon with respect to $v_{\ell }$. □

### Lemma 12

The curve *C* has no rational points at infinity.

### Proof of Lemma 12

This follows from the leading coefficient of $f_{S,\mathcal {B}}(X)$ having ℓ-adic valuation equal to 2. □

We now want to show $C({\mathbb {Q}})$ is exactly *S*. In other words, we want to show that if $f_{S,\mathcal {B}}(x)=y^d$ for some $x,y\in {\mathbb {Q}}$, then (x,y)∈S. We argue using *p*-adic valuations, beginning with the following technical Lemma.

### Lemma 13

Let $x\in {\mathbb {Q}}$ and r>1. Then $v_p(g(x))<0$ if and only if $v_p(x)<0$.

### Proof of Lemma 13

First, let *p* be a prime such that $v_p(g(x))<0$. Then

$$\begin{aligned} v_p(g(x))=v_p(\ell )+6v_p( N)+6\sum _{i=1}^r v_p(x-a_i)&<0. \end{aligned}$$

Since $0\le v_p(\ell N) \le 1$ we must have that $\sum v_p(x-a_i)<0$. As the $a_i$ are all integral, we have $\sum v_p(x-a_i)=rv_p(x)<0$, so $v_p(x)<0$.

Conversely, if $v_p(x)<0$, then

$$\begin{aligned} v_p(\ell )+6v_p( N)+6\sum _{i=1}^r v_p(x-a_i)=v_p(\ell )+6v_p(N)+6rv_p(x)&<0. \end{aligned}$$

since r>1, so $$v_p(g(x))<0$$. □

We use this Lemma 13 to show $f_{S,\mathcal {B}}(X)=0$ has no rational solutions.

### Lemma 14

$f_{S,\mathcal {B}}(X)$ has no rational roots.

### Proof of Lemma 14

Suppose there exists $x\in {\mathbb {Q}}$ such that $f_{S,\mathcal {B}}(x)=0$. Note that g(x)=0 is not possible since g(x)≥1 for all $x\in {\mathbb {R}}$, so we must have that (h(x)-1)g(x)+1=0, and so

4 $$\begin{aligned} v_p(h(x)-1)+v_p(g(x))=0. \end{aligned}$$

We show that for all *p* we have $v_p(h(x)-1)\ge 0$.

If $v_p(g(x))\le 0$ then by (4) we have that $v_p(h(x)-1)\ge 0$.

If $v_p(g(x))> 0$, then by Lemma 13 we have $v_p(x)\ge 0$. If $v_p(m)>0$ then $v_p(h(x)-1)=v_p(g(x))=0$ by our choice of *N*, so $v_p(h(x)-1)=0$. Therefore assume $v_p(m)=0$. Then we have that $v_p(h(x))=v_p(mh(x))$. Since $v_p(x)\ge 0$ and $mh(X)\in {\mathbb {Z}}[X]$, we have that $v_p(mh(x))\ge 0$, so $v_p(h(x))\ge 0$, from which we have that $v_p(h(x)-1)\ge 0$.

Since for all *p* we have $v_p(h(x)-1)\ge 0$, this means $h(x)-1\in {\mathbb {Z}}$. Since g(x)≥1 and (h(x)-1)g(x)=-1 we must have g(x)=1, so $x=a_i$ for some *i*. Since *S* is acceptable, and $f_{S,\mathcal {B}}(a_i)=b_i^d$, we reach a contradiction.

□

### Lemma 15

$$C({\mathbb {Q}})=S$$.

### Proof of Lemma 15

By Lemma 12 we may restrict to the affine patch. Suppose $(x,y)\in C({\mathbb {Q}})$. Then we have that

$$\begin{aligned} f_{S,\mathcal {B}}(x)=y^d. \end{aligned}$$

By Lemma 14 we know that y≠0. Henceforth suppose $(x,y)\in C({\mathbb {Q}})$ and y≠0. We will start by showing that this implies $v_p(g(x))\in 3{\mathbb {Z}}$ for all primes *p*, where

$$\begin{aligned} g(x)=\ell N^6 \prod _{i=1}^r(x-a_i)^6+1. \end{aligned}$$

First, let *p* be a prime such that $p\ne \ell$ and $v_p(g(x))<0$. Then we see that

$$\begin{aligned} v_p(g(x))=6v_p( N)+6\sum _{i=1}^r v_p(x-a_i)&\in 3{\mathbb {Z}}. \end{aligned}$$

Now, let *p* be a prime such that $p\ne \ell$ and $v_p(g(x))> 0$. By Lemma 13 we must have that $v_p(x)\ge 0$. If $v_p(m)>0$ then $v_p(g(x))=0\in 3{\mathbb {Z}}$ by our choice of *N*, so we can assume $v_p(m)=0$. Then we have that $v_p(h(x))=v_p(mh(x))$. Since $v_p(x)\ge 0$ and $mh(X)\in {\mathbb {Z}}[X]$, we have that $v_p(mh(x))\ge 0$, so $v_p(h(x))\ge 0$. Since $v_p((h(x)-1)g(x))>0$ we conclude that

$$\begin{aligned} v_p((h(x)-1)g(x)+1)=0. \end{aligned}$$

Recall $$f_{S,\mathcal {B}}(x)=y^d$$, so

$$\begin{aligned} v_p(g(x))=v_p\left( g(x)((h(x)-1)g(x)+1)\right) \equiv 0\mod d \end{aligned}$$

and since 3∣d, we see that $v_p(g(x))\in 3{\mathbb {Z}}$.

Now, consider $v_{\ell }(g(x))$. If $v_{\ell }(x)\ge 0$, then $v_{\ell }(g(x))=0\in 3{\mathbb {Z}}$. So suppose $v_{\ell }(x)<0$. Recall we have chosen ℓ to satisfy the conditions of Proposition 10, and in particular condition (10) by Lemma 9. Our choice of ℓ from Lemma 9 guarantees that $v_{\ell }(\alpha _n)=0$, where $\alpha _n$ is the leading coefficient of *h*(*X*). From this, it follows that

$$\begin{aligned} v_\ell (h(x)-1)=nv_{\ell }(x). \end{aligned}$$

As before we have that $v_{\ell }(g(x))=1+6rv_{\ell }(x)$, and so

$$\begin{aligned} v_\ell \left( g(x)((h(x)-1)g(x)+1)\right) =2+dv_{\ell }(x)\equiv 0 \mod d, \end{aligned}$$

which is not possible. So $v_\ell (x)\ge 0$ and $v_\ell (g(x))=0\in 3{\mathbb {Z}}$.

Since $v_p(g(x))\in 3{\mathbb {Z}}$ for all primes *p*, we have shown that $g(x)=\beta ^3$ for some $\beta \in {\mathbb {Q}}^\times$. In other words, the following expression holds,

$$\begin{aligned} \ell \left( N^3 \prod _{i=1}^r(x-a_i)^3\right) ^2=\beta ^3-1. \end{aligned}$$

This implies there is a rational point $(\beta ,\gamma )$, where $\gamma :=N^3\prod _{i=1}^r(x-a_i)^3 \in {\mathbb {Q}}$, on the elliptic curve

$$\begin{aligned} E_{\ell }:\ell Y^2=X^3-1, \end{aligned}$$

with $\ell \equiv 5 \mod 12$. By Theorem 16, $E_{\ell }({\mathbb {Q}})=\{(1,0),\infty \}$. Since $\beta \ne 0$, the point $(\beta ,\gamma )$ is not the point at infinity, so we conclude that $(\beta ,\gamma )=(1,0)$. Therefore g(x)=1 and $x=a_i$ for some *i*. Then

$$\begin{aligned} f_{S,\mathcal {B}}(x)=f_{S,\mathcal {B}}(a_i)=b_i^d, \end{aligned}$$

and since *d* is odd, we conclude that $y=b_i$. □

### Theorem 16

[8, Theorem 2] Let $E:Y^2=X^3-1$ be the Mordell curve, and let

$$\begin{aligned} E_{p}:pY^2=X^3-1 \end{aligned}$$

denote its twist by a prime *p*. Then for all primes $p\equiv 5 \mod 12$, we have

$$\begin{aligned} E_{p}({\mathbb {Q}})\cong \{(1,0), \infty \}. \end{aligned}$$

### Proof of Theorem 5

Let $S=\{(a_1,b_1),\dots ,(a_r,b_r)\}\subset {\mathbb {A}}^2({\mathbb {Q}})$ be the given acceptable set. We have already shown in (2) that $S\subset C({\mathbb {Q}})$. Lemma 15 shows that $C({\mathbb {Q}})\subset S$, so $C({\mathbb {Q}})=S$ as required. The polynomial $f_{S,\mathcal {B}}$ is separable by Lemma 11. □

## Data Availability

Data sharing is not applicable to this article as no datasets were generated or analysed in the current paper.
